# Effects of topical gel formulation of *Ficus carica* latex on cutaneous leishmaniasis induced by *Leishmania major* in BALB/c mice

**DOI:** 10.1186/s13104-021-05614-8

**Published:** 2021-05-22

**Authors:** Ali Pouryousef, Erfan Eslami, Sepehr Shahriarirad, Sina Zoghi, Mehdi Emami, Mohammad Reza Cheraghi, Bardia Zamiri, Soliman Mohammadi-Samanii, Bahador Sarkari

**Affiliations:** 1 Department of Parasitology and Mycology, School of Medicine, Shiraz University of Medical Sciences, Shiraz, Iran; 2 Student Research Committee, School of Medicine, Shiraz University of Medical Sciences, Shiraz, Iran; 3 School of Pharmacy, Shiraz University of Medical Sciences, Shiraz, Iran; 4 Basic Sciences in Infectious Diseases Research Center, Shiraz University of Medical Sciences, Shiraz, Iran

**Keywords:** *Ficus carica*, Latex, Cutaneous leishmaniasis, *Leishmania major*

## Abstract

**Objectives:**

The current study aimed to evaluate the effects of *Ficus carica* latex on the treatment of cutaneous leishmaniasis (CL), induced by *Leishmania major.* A 5% topical gel with *F. carica* latex was prepared. BALB/c mice were infected by inoculation of amastigotes form of *L. major.* Thirty BALB/c mice were divided into five groups, where the first group was treated daily, the second group twice per day, and the third group every other day with the 5% topical gel, for 3 weeks. The sizes of the lesions were measured before and during the course of treatment.

**Results:**

Although the mean size of lesions in the mice group treated with the 5% *F. carica* gel, especially in the group receiving daily treatment, was less than the mean size of the lesions in the control group, yet, the differences was not statistically significant (p > 0.05). The findings of the current study demonstrated that the 5% *F. carica* latex with a 3-week course of treatment had no considerable effect in recovery or control of CL induced by *L. major* in the murine model. Using higher concentration of *F. carica* latex and with longer treatment lengths may increase its efficacy in the treatment of CL.

## Introduction

Despite the great advances in the diagnosis and treatment of cutaneous leishmaniasis (CL), the disease is still one of the most important health challenges in many countries around the world.

With diverse clinical manifestations, CL is endemic in many countries in the Americas, the Mediterranean Basin, Middle East, and Central Asia [1–6]. Approximately 90% of CL cases occur in Iran, Afghanistan, Algeria, Iraq, Saudi Arabia, Syria, Brazil, and Peru. In Iran, both visceral and cutaneous forms of the disease are present [4, 5, 7–9]. Treatment of CL remains a challenge and the pentavalent antimonials, which are still the mainstay for treatment of CL, are relatively toxic, require many injections and are not always effective [10–12]. Therefore, finding new compounds for the treatmentof this disease seems necessary. In traditional medicine several native plants have been used for the treatment of CL [13, 14]. Among these are garlic, shallots, wormwood, yarrow, walnuts, thyme, henna plant, mimosa, aloe, which are having, to some extent, anti-leishmanial effects [13].

Ficus plants belongs to *Moraceae* family and consist of extremely different plants. *Ficus carica* is an Asian species of fig, well-known as the common fig. The fig fruit is considered to be beneficial in traditional medicine in the treatment of various diseases including diabetes, warts, cancer, and gastrointestinal disorders [15]. The latex of the plant is milky white and is used, by several indigenous communities, as a curdling agent in the production of milk product like cheese and yoghurt [16]. Numerous bioactive compounds including phenolic compounds, namely, psoralen and bergapten, phytosterols, free amino acids, saturated and unsaturated fatty acids, phytosterols, and cysteine are present in the *F. carica* latex. Besides, the fig’s latex is a rich source of proteolytic enzymes, among them is ficin S which is a well characterized sugar containing proteinase. Ficus latex is also reported to have several other enzymes, including lipase, catalase, peroxidase, protease, rennin, diastase, and esterase [16–19].

The latex of fig shows antioxidant, antifungal, chitinolytic, cytotoxic, antiviral, antibacterial and anthelmintic activities [15, 20–25].The active compounds of Ficus latex responsible for its antimicrobial activity has been reported in some studies. It has been shown that the chlorophormic, ethyl acetate, methanol, and hexanoic extracts of latex has antifungal activities against *Aspergellus fumigates*, *Trichophyton rubrum*, *Trichophyton soudanense*, *Microsporum canis*, *Scopulariopsis brevicaulis*, *Candida albicans*, and *Cryptococcus neoformans* [15]. The antifungal property of *F. carica* latex has been attributed to its chitin-binding protein [26]. Two natural furocoumarins of *F. carica* latex, 5-methoxypsoralen and 8-methoxypsoralen, has shown significant toxic effect against larvae of *Aedes aegypti* [27]. Moreover, cysteine proteinases of *F. carica* latex are known to be significantly effective against rodent intestinal nematodes, including *Heligmosomoides polygyrus* and *Trichuris muris* [28]. Also, the anthelmintic activity of *F. carica* latex against *Syphacia obvelata*, have been documented [22].

Considering the antibacterial and antifungal properties of *F. carica* latex, the current study aimed to evaluate the anti-leishmanial effects of *F. carica* latex for the treatment of CL, induced by *L. major* in BALB/c mice.

## Methods

### Preparation of *F. carica* latex gel

The latex of *F. carica* was obtained from the fig tree in summer, 2020, in Fars Province, southern Iran. Fifty mL of the latex was freeze-dried which was somewhat gelatinous. To prepare a 5% gel of *F. carica* latex, the lyophilized latex was levigated with Eucerin in a 1:9 weight ratio, according to the geometric method, and homogenized following by mixing with an equal volume of petrolatum. The final product was kept in an adequate jar in a dark and cool place until use. The choice of latex concentration was based on the fact that in previous studies on the effect of fig latex on other microorganisms including fungi and viruses, these concentrations were considered to be effective.

### Inoculation of BALB/c mice with *Leishmania* parasites

Thirty inbred female BALB/c mice (4-week-old, 25–30 g body weight) were obtained from Pasteur Institute, Tehran, Iran. The animals were kept in condition with a 12:12-h dark–light cycle, 40–50% humidity, at 25 ± 3 °C temperature. Free access to water and a standard diet was provided for the animals. *L. major* (MRHO/IR/75/ER) was used in this study. Mice infection was done by subcutaneous inoculation of amastigotes form of *L. major*, (0.2 mL of a suspension containing 4 × 10^6^ amastigotes) into the top of mice's tail base by insulin syringe. The cutaneous lesions were developed after about 4 weeks of inoculation.

### Treatment with *F. carica* latex topical gel

The thirty BALB/c mice were divided into five groups (six mice in each group). The first group received 5% gel, daily, the second group received the gel twice a day, and the third group received the gel every other day. The duration of treatment in all groups was 3 weeks. The positive and negative controls received amphotericin B (intramascular, daily) and the gel base, respectively. The size of the lesion was measured with a direct reading digital Vernier caliper, before and during the course of treatment. At the end of the treatment period, a skin sample from the lesion area was taken and evaluated for the parasite load, using the methods described by Ezatpour et al. [29]. Briefly, lesions were cleaned with 70% ethanol and smears were prepared by the sterile lancet, fixed by methanol, and stained with Giemsa stain. The load of parasites was determined, considering the following guideline. Negative: 0 parasite/10 fields, 1 + : 1 parasite/10 field, 2 + : 1–10 parasites/10 fields, 3 + : 10–100 parasites/10 fields, 4 + : 1010–1000 parasites/10 fields, 5 + and more: more than 1000 parasites/10 fields. The animals were euthanized at the end of experiment, using ketamine–xylazine (100 µL of a 10:1 ketamine–xylazine solution, IP).

### Statistical analysis

The mean sizes of the lesions in the five groups of mice were analyzed by SPSS (ver. 20). Nonparametric Kruskal Wallis test was used to find out the mean differences of the size of lesions in the five groups of studied mice. The pre-intervention measures (time = 0, before treatment) were used for adjustment of trend and grouping comparison. Significance was established for a P-value of < 0.05.

## Results

The mean sizes of leishmanial lesions during the 3 weeks of the study for different treatment regimens of the 5% *F. carica* gel and the negative and positive control groups are presented in Table 1. Although the mean size of lesions in the mice group treated with the 5% *F. carica* gel, especially in the group receiving daily treatment, was less than the mean size of the lesion in the control group, yet, the differences were not statistically significant (p > 0.05). The post-hoc comparison suggests that there is a difference in favor of daily treatment with 5% gel, as the most effective treatment regimen, yet the difference was not statistically significant (P > 0.05). Direct smears of lesions in the microscopic investigations were positive for the *Leishmania* amastigotes, after 3 weeks of treatment in all control and experimental groups. The microscopic examination of the smears prepared from the mice skin lesions showed a lower parasite load in the positive control group and the gel receiving group in comparison with the negative controls.

**Table 1:** Mean sizes of CL lesions (mm) in BALB/c mice before and during the treatment with *F.carica* latex gel

Group of mice under treatment	Before treatment	First week	Second week	Third week	p-value
Positive control	6.57 ± 0.85	6.31 ± 0.69	6.3 ± 0.78	6.28 ± 1.05	0.073
Negative control	7.74 ± 1.07	7.86 ± 0.93	8.12 ± 1.2	8.27 ± 1.16	0.125
Test (once daily)	6.5 ± 1.08	6.6 ± 1.41	6.37 ± 1.28	6.45 ± 1.33	0.152
Test (twice daily)	5.73 ± 0.81	6.1 ± 1.36	6.3 ± 2.16	6.35 ± 2.56	0.168
Test (every other day)	6.8 ± 0.35	6.79 ± 0.38	6.78 ± 0.84	6.79 ± 1.13	0.085

All data are presented in mean ± SD

## Discussion

Pentavalent antimony compounds remain the drug of choice for the treatment of CL as well as other forms of leishmaniasis. Nevertheless, treatment with antimony agents has been associated with several challenges, including toxicity and drug resistance [10]. Therefore, finding new compounds for the treatment of leishmaniasis is a serious necessity that has attracted the attention of many researchers. In the current study, the anti-leishmanial effect of *F. carica* latex, on *L. major* was evaluated. Results of the study demonstrated a reduction in lesion sizes in BALB/c mice treated daily with the 5% gel of the latex, but the differences were not statistically significant when compared with the control groups. The findings indicated that the 5% concentration *F. carica* latex in topical gel formulation has no substantial effects on inhibiting the growth of the parasite. These findings are in agreement with the study of Saleem et al. in which the aqueous extract of *F. carica* had weak in vitro activity against the intracellular form of both *L. donovani* and *L. major* [30]. The low concentration of latex and short-term treatment period might play an important role in obtaining such a result in our study. This means that preparing a gel with a higher concentration or extending the duration of treatment may have better results. However, higher concentration of latex may have toxicity for the host as documented by Alziro et al. where they showed that the *F. carica* latex in a dose of 3 ml/kg/day had a high toxicity effect on studied mice [22].

Another reasons of inefficiency of fig’s latex in the treatment of CL in the present study can be attributed to the harvesting time of fig’s latex, as the latex has different levels and compositions at different times of plant and fruit growth. Support from this notion comes from Raskovic et al., study which reported that the highest chitinolytic activity of the fig’s latex is at the beginning of flowering and the highest antifungal activity is in spring [31].

Another concern about the application of fig’s latex is the irritating properties of its compounds, which have been reported in various studies. The irritating effects of fig latex is linked to the presence of compounds such as calotropenyl acetate, methyl maslinate, and lupeol acetate in it [32]. However, in the present study, no apparent irritating effect was observed in the studied mice. This may be due to the relatively low concentration of the used latex in this study and the dissolution of the irritating lipid compounds in the petrolatum, used for the preparation of the gel.

In the present study, the sizes of leishmaniasis lesion in positive control mice before and after treatment did not show a significant difference. This may be due to the resistance of the strain to the drugs which has been used in the study in the treatment of CL.

## Conclusion

The 5% concentration of *F. carica* latex had no considerable effect in the treatment of CL induced by *L. major* in the BALB/c mice. Further studies, using higher doses of *F. carica* latex and with longer treatment lengths may increase the efficacy of *F. carica* latex in the treatment or control of CL. In addition, better results may be achieved by preparation of latex in different plant growth seasons.

### Limitations

There are several limitations to this study. First, there was no improvement in the positive control mice despite treatment. Second, the gel was used in a limited dose. Lastly, the length of treatment could be longer.

## Data Availability

Any further requested information regarding the experimental and data analysis during the current study is available from the corresponding author on request.

## Abbreviation

CL: Cutaneous leishmaniasis
